# XRD and Spectroscopic Investigations of ZIF—Microchannel Glass Plates Composites

**DOI:** 10.3390/ma16062410

**Published:** 2023-03-17

**Authors:** Justin Narimbi, Sivakumar Balakrishnan, Tatiana S. Perova, Garret Dee, Gerhard F. Swiegers, Yurii K. Gun’ko

**Affiliations:** 1 Department of Applied Sciences, The PNG University of Technology, Lae MP 411, Morobe Province, Papua New Guinea; 2 Department of Electronic and Electrical Engineering, Trinity College Dublin, The University of Dublin, D02 PN40 Dublin, Ireland; 3 School of Chemistry, Trinity College Dublin, The University of Dublin, D02 PN40 Dublin, Ireland; 4 Intelligent Polymer Research Institute, University of Wollongong, Wollongong, NSW 2522, Australia

**Keywords:** ZIF-L, ZIF-8, ZIF-67, microchannel glass (MCG) plate, hydrothermal method

## Abstract

In this study, new composite materials comprising zeolitic imidazolate framework (ZIF) structures and microchannel glass (MCG) plates were fabricated using the hydrothermal method and their morphological and spectral properties were investigated using XRD, SEM, FTIR, and Raman spectroscopy. XRD studies of powder samples revealed the presence of an additional phase for a ZIF-8 sample, whereas ZIF-67 samples, which were prepared through two different chemical routes, showed no additional phases. A detailed analysis of the FTIR and micro-Raman spectra of the composite samples revealed the formation of stable ZIF structures inside the macropores of the MCG substrate. The hydrophilic nature of the MCG substrate and its interaction with the ZIF structure resulted in the formation of stable ZIF-MCG composites. We believe that these composite materials may find a wide range of important applications in the field of sensors, molecular sieving.

## 1. Introduction

Porous nanomaterials have received considerable attention in the recent past due to their numerous useful applications [1,2]. Such nanomaterials include metal–organic frameworks (MOFs). MOFs have extraordinary tunable and flexible structural properties [3,4] owing to the organic ligands and metal nodes that make up these materials. Due to the way ligands are bonded to metal nodes, MOFs are often referred to as “special materials” which have very unique properties, such as a high surface area, high porosity, adjustable pore sizes, and flexible functionality, thus offering great potential for a multitude of applications [5]. 

Recent reports have demonstrated that MOFs are increasingly being studied for gas storage and separation, catalysis, magnetism, luminescence, sensor technologies, drug storage and delivery, electrochemical charge storage technologies, and environmental pollution-abatement applications [6,7,8,9,10,11].

ZIFs (zeolitic imidazolate frameworks) are a subclass of MOF materials [12] and provide an easily adjustable pore size and surface functionality, whilst demonstrating high thermal and chemical stability with an interconnected three-dimensional structure [13]. In addition, the active site on the surface of the ZIF compound can behave both as an acid and as a base, depending on the specific interaction of the site with a metal ion, such as Zn, Cu, or Co, and imidazole groups [14]. Interaction (coordination) between metal ions and imidazole linkers (metal-Im-metal) forms a tetrahedral topology with similar bonding angles to that of Si-O-Si in natural zeolites. Among the investigated ZIF materials, ZIF-8 has unique advantages in terms of its chemical and thermal stability [1,15]. Furthermore, many studies reference ZIF-8 in relation to environmental remediation, since ZIF-8 has advantages over traditional approaches, in terms of manufacturability and cost [16,17,18,19,20]. While ZIF materials find use in powder form, powder alone is not suitable for many applications because powder materials are fragile and brittle. To overcome this shortcoming, researchers have used porous or nonporous supports to enable viable end-application [21,22,23]. In particular, porous substrates, such as AAO, alpha alumina, ceramic hollow fibre substrates, and MCG can provide additional support and strength. ZIF-based membranes, with nanometre to angstrom level pore diameters, can potentially differentiate the selective movement of chemical moieties through the membrane. Thus, it could be possible to electrochemically separate small molecules from larger ones.

With regard to optical applications, recent publications have reported on the unique linear and nonlinear optical properties of ZIFs as well as of various ZIF-based composite systems. In particular, in [24] a very large change in the refractive index (with Δ*n* ≈ 0.20) has been demonstrated using porous ZIF-8 as the host structure impregnated with various organic guest molecules. This allows the use of ZIF composites for sensors, fibre optic communications, and optical data storage applications. In addition, for ZIF-8 [25,26] and ZIF-67 [27,28], a second-order nonlinear optical (NLO) response was demonstrated over a wide optical range (visible and near-infrared), while in [29] for ZIF-8, a third-order nonlinearity demonstrated enhancement with increasing film thickness. Currently, commercial NLO materials are mostly inorganic crystals, however, organic materials have clear advantages in terms of manufacturability, tunability and response time. NLO properties are important for ZIF applications in laser generation, electro-optical switching, optical computing, and telecommunications.

In addition, the recent realization of ZIFs as a promising alternative material for use in membrane separation, gas enrichment, and purification applications has prompted an increase in ZIF materials research [13]. For example, Sutrisna et al. [30] reported on the increasing attention given to ZIF-8 membranes and ZIF-8 membranes fabricated on polymer substrates. Commonly used approaches to grow ZIF, or MOF in general, on porous support materials are in situ growth, electrochemical synthesis, anodic oxidation, or the secondary seeding method [23]. In situ growth is a one-step process in which the substrate is dipped into a ZIF precursor solution without any prior seeding process. The nucleation, growth, and intergrowth of a crystal on the substrate material can then occur simultaneously in the precursor solution [31]. Both inorganic and organic support materials have been used to fabricate ZIFs with some success, although not without difficulty, especially with the use of organic polymeric substrates.

The use of mixed-matrix membranes has, to some extent, overcome the challenges associated with the use of organic polymeric substrates [31]. However, one of the generally accepted methods for growing MOFs or ZIFs on substrates is to use substrates functionalised with silicon dioxide or OH [32] or by seeding the substrate with silicalite material prior to ZIF growth [33]. The method is effective because of the interaction of the surface silanol groups with the MOFs or ZIFs, which facilitates the fixation of the MOFs/ZIFs on the surface of the substrates. Tu et al., reported the direct growth of ZIFs on a silica-coated substrate and emphasised the importance of a thermally stable interface [32].

The growth of ZIFs on porous substrates offers significant advantages over their nonporous counterparts, since the internal morphology of support materials can provide protection against damage caused during handling (mechanical and physical) [34]. Moreover, ZIFs fabricated on flat supports are of limited use on an industrial scale due to their low packing density and low area-to-volume ratio [35]. However, several physical properties may affect the performance of hollow support materials which have ZIFs grown on the inner surfaces, as described by Huang et al. [34], specifically, pore size and structure, surface chemical composition, and thermal and chemical stability. Another challenge is to achieve successful deposition of ZIF crystals inside the cavity of the hollow substrate material, consistent with that of the outer surfaces [34].

The most common approach to obtain ZIFs is solvothermal/hydrothermal synthesis, however, alternative approaches have been reported, including microwave, sonochemical, mechanochemical, dry-gel conversion, solvent-free oxide/hydroxide based, microfluidic, and electrochemical methods [36]. Li et al. [13] used seven different synthesis methods, including conventional solvothermal dimethylformamide (DMF), solvothermal methanol (MeOH), microwave, sonochemical, mechanochemical, dry-gel conversion (DGC), and microfluidic methods to achieve successful ZIF synthesis. However, all of these methods require either expensive instruments or complex experimental procedures. Additionally, the preparation of a seeded material inside the porous substrate requires additional materials to enable the growth of the seed material, which is often time-consuming. For these reasons, our study focuses on ZIF growth on a microchannel glass (MCG), which has the functional group required for ZIF anchoring. MCG materials are thin, planar structures made of glass, characterised by high aspect ratio pores which run perpendicular to the substrate plane [37]. MCG has an open area ratio of 60% or more, with thickness usually in the range 0.5 mm to 1.5 mm and pore diameters of approximately 10 μm (as in this study). Our work demonstrates the preparation of ZIF–MCG composites (hereinafter referred to as ZIF@MCG) using conventional solvothermal methanol or water-based methods. For the preparation of ZIF-67, two methods were adopted which differ mainly in the solvents used. For example, the first method used methanol, and the second method used deionised (DI) water, along with a surfactant. Our method takes into account the hydrophilic nature of the MCG substrate and its interaction with ZIF precursors, promoting the formation of stable ZIF structures. The composites produced have been characterised using X-ray diffraction (XRD), Raman microscopy, and Fourier transform infrared spectroscopy (FTIR).

## 2. Materials and Methods

### 2.1. Synthesis of ZIF-8 and ZIF-8@MCG Composite

ZIF-8 was synthesised according to the previously described procedure with some modifications [38]. Briefly, 1 mmol Zn(NO_3_)_2_.6H_2_O was dissolved in 2.5 mL methanol (MeOH) in a scintillation vial. The ligand, 8 mmol of 2-methylimidazole (2-MeIm), was then dissolved in 2.5 mL of MeOH in a separate small beaker. The 2-MeIm solution was slowly added to the zinc nitrate solution. The solution was then heated in a Teflon-lined Parr digestor at 150 °C for 5 h. The vessel was then cooled to room temperature, the yellowish solution was carefully removed, and the crystals were washed (twice) with methanol. ZIF-8 crystals were dried at room temperature and stored in a desiccator. The ZIF-8-microchannel glass composite was prepared using the same method as the ZIF-8 powder. The MCG was placed in a solution containing a mixture of zinc nitrate and ligand before being heated in a Teflon-lined Parr digester. After cooling to room temperature, the MCG was removed, washed twice with methanol, and dried at room temperature in a desiccator.

### 2.2. Synthesis of ZIF-67 and ZIF-67@MCG Composite

Two methods were adopted for the synthesis of ZIF-67 and the ZIF-67@MCG composite (herein referred to as route A and route B). The ZIF-67 obtained using routes A and B is hereinafter referred to as ZIF-67-1 and ZIF-67-2, respectively. Procedures for the synthesis of ZIF-67 were taken from the literature with minor changes [39,40].

#### 2.2.1. Synthesis of ZIF-67-1 and ZIF-67-1@MCG Composite (Route A Method)

First, 2.5 mmol Co(NO_3_)_2_.6H_2_O was dissolved in 50 mL of MeOH. This was solution A. Next, 20 mmol 2-MeIm was dissolved in a separate solution of 50 mL MeOH with stirring (solution B). Solution A was then poured into solution B while stirring. The mixture was stirred continuously for 18 h at room temperature. The precipitate was recovered by centrifugation. The precipitates were washed three times with 10 to 15 mL of MeOH and recovered by centrifugation after each washing. The crystals were dried at 60 °C overnight and stored in scintillation vial for later use. For the synthesis of ZIF-67@MCG composite using this route, the same procedure was adopted but with the MCG suspended in the ZIF-67 precursor solution with the support of a wooden peg at one end which was clamped to a retort stand. After 18 h of continuous stirring, the MCG was carefully removed, washed three times with 10 to 15 mL MeOH and dried in oven at 60 °C. It was then stored in a desiccator. 

#### 2.2.2. Synthesis of ZIF-67-2 and ZIF-67-2@MCG Composite (Route B Method)

For route B, 6 mL of deionised water (Milli-Q) was taken and 174 mg of Co(NO_3_)_2_.6H_2_O and 3 mg of cetyltrimethylammonium bromide (CTAB) were added. The mixture was stirred to obtain a clear solution. This was solution A. In a separate clean beaker, 2.7 g of 2-MeIm was dissolved in deionised water. This was solution B. Solution A was quickly added to solution B while stirring and stirred for 20 min. The mixture was centrifuged, washed three times with 10 mL of ethanol (EtOH) and the precipitates were dried in an oven at 60 °C overnight. The dried crystals were transferred to a scintillation tube and stored for later use. The same procedure was adopted for the synthesis of the ZIF-67-2@MCG composite. Here, the MCG was suspended in the precursor solution in the same manner as previously described for the ZIF-67-1 synthesis. The MCG was carefully removed, washed three times with 10 mL EtOH, dried in an oven at 60 °C overnight and stored in a desiccator.

Information on all fabricated samples used for further investigations is summarised in Table S1.

### 2.3. Characterizations of ZIF-8, ZIF-67, and Their Composites

#### 2.3.1. XRD Measurements

X-ray powder diffraction was carried out using a Bruker, D2 Phaser 2nd generation, powder XRD (PXRD) machine with a copper X-ray source of wavelength 0.154 nm. All scans were over the range 15–85° 2 θ and were run for 2.2 h unless otherwise stated. The results were processed with Bruker Diffrac.Eva software and Panalytical HighScore Plus software as needed.

#### 2.3.2. Micro-Raman Spectroscopy

Raman measurements were carried out in backscattering geometry using a Renishaw 1000 micro-Raman system with a motorised positional stage and Leica microscope. A He-Ne laser at wavelengths of 633 nm at a power of <10 mW (at the laser output) was used as the excitation source. The power was kept low to prevent the sample overheating. To reduce power further, laser filters were used with 50% of transmittance from the original laser power. The laser spot was focused on the surface of the microchannel glass in case ZIF infiltrated inside the pores using 50× magnification objectives with a short-focus working distance (see Figure S1). Spectra were also collected by focusing on the pores from both sides of the MCG (Figure S1). In the case of the powder samples, which were deposited onto a cleaned silicon wafer, the laser spot was focused on small pieces of powder, using 50× magnification objectives with a long-focus working distance. Since the angle of the scattering light cone with this objective is smaller than one obtained with short-focus working distance objectives, the registered spectra, even at a long time of signal accumulation (100 s), were relatively weak.

#### 2.3.3. FTIR Spectroscopy

FTIR spectra for investigated samples were registered on a FTIR spectrometer Perkin Elmer Spectrum 100 using universal attenuated total reflection (UATR) accessories with a diamond ATR prism. Spectra were collected in the region of 280–4000 cm^−1^ with a resolution of 1 cm^−1^ and a scanning time of 3 min. 

## 3. Results and Discussion

### 3.1. XRD Analysis

**Figure 1:**
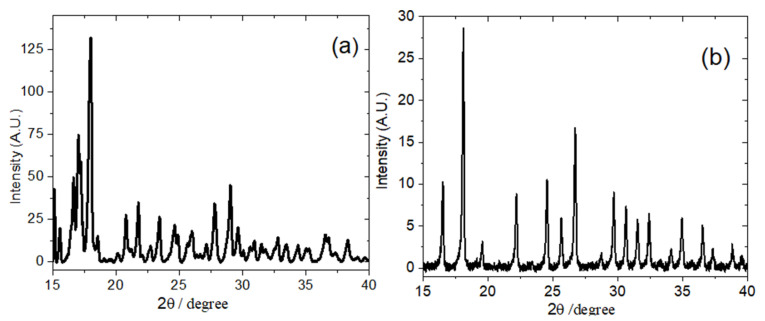
XRD pattern of (**a**) ZIF-L/-8 (jn-01), and (**b**) ZIF-67-1 (jn-04) powder samples.

Figure 1a,b shows XRD patterns of the as synthesised ZIF-8 (presumably ZIF-L) and ZIF-67-1 powder samples. For the ZIF-67-1 sample, the peaks had an excellent match with the COD 4118891 database card, confirming that ZIF-67, Co(mIm)_2_, had been synthesised. ZIF-67-1 demonstrated excellent agreement with the previously published ZIF-67 material with the main peaks at index as 7.9° (011), 10.6° (002), 12.9° (112), 14.7° (022), 16.5° (013), 18.4° (222), 22.0° (114), 24.6° (233), 25.9° (002), 26.4° (134), 29.6° (044), 31.2° (244), and 32.3° (235) [41]. This ZIF phase corresponds to the SOD (sodalite-like) topology with high surface area [42].

The ZIF-8(ZIF-L) sample gave a match with the COD 7222297 database card, which is an intermediate phase corresponding to the ZIF-L morphology with the main peaks index as 7.3° (200), 7.7° (111), 10.4° (020), 10.9° (112), 11.5° (202), 12.7° (220), 12.9° (311), 13.5° (221), 13.7° (022), 15.1° (312), 15.6° (222), 16.7° (131), 17.0° (023), 17.2° (402), 18.0° (420), 21.7° (314), 27.7° (135), and 29.0° (443) [43]. Previous work has demonstrated that the ZIF-L phase can easily be transformed into the ZIF-8 phase Zn(mIm)_2_ by heating it in ethanol at 60 °C for 72 h [44]. Recent studies have demonstrated the instability of ZIF-8 in aqueous environments and the authors in [45] have attributed the main reason for contradictory reports on ZIF-8 stability to the sample collection method. 

A comparison between XRD patterns for sample jn-01 before and after heating at 65 °C for 150 h is shown in Figure S2a,b. This is followed by a brief discussion on the EDX and SEM data obtained which are also included in the SM section. Included SEM images are that of samples jn-02 and jn-06 and these are shown in Figure S3a,b.

### 3.2. FTIR Spectra

**Figure 2:**
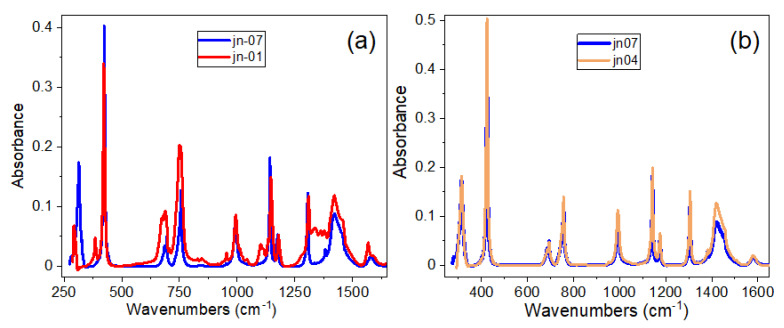
Comparison of FTIR spectra in powder form for samples (**a**) jn-01 and jn-07 and (**b**) for samples jn-07 and jn-04.

The FTIR spectra of ZIF-L/-8 and ZIF-67, synthesised by two different routes, namely ZIF-L/-8 (jn-01) and ZIF-67-2 (jn-07), are shown in Figure 2a,b. A comparison between the FTIR spectra of jn-01 and jn-07 samples (shown in Figure 2a) demonstrates some small deviation in peak position and their relative intensity which are in good agreement with the published data on these materials [46,47,48,49]. In brief, most of observed vibrational bands are associated with the vibrations of imidazole (Im) units with the exception of Zn-N stretching, located at 421 cm^−1^. In the region 600–800 cm^−1^, the vibrational bands are associated with the out-of-plane imidazole ring puckering (bending), while in-plane bending modes appeared in the region 900–1350 cm^−1^. The complicated composite band in the region 1350–1500 cm^−1^ is assigned to entire ring stretching, the peak observed at 1585 cm^−1^ is related to C=N stretching mode, whereas bands at 2927 and 3135 cm^−1^ are attributed to the aromatic and aliphatic C-H stretching of Im, respectively [46,47,48,49].

At the same time, as seen in Figure 2b (also shown in more detail in Figure S4), there is no significant difference in the FTIR peak positions and intensities for the ZIF-67 samples (namely ZIF-67-1 and ZIF-67-2). This demonstrates that the preparation routes have not affected the peak positions of the ZIF-67 samples and the structural equality in samples prepared through these two routes is maintained. 

Although the X-ray diffraction patterns for ZIF-8 and ZIF-L are drastically different, it is not so easy to confirm one or another structure using FTIR spectroscopy, since both structures have similar chemical bonds resulting in a small deviation in peak positions and their relative intensities. Based on some of the specific peaks described in the literature as being unique to ZIF-L, such as 1771 and 2426 cm^−1^ [44], we cannot definitively conclude whether this structure is ZIF-8 or ZIF-L. Both of these peaks did not appear either in the IR spectrum of the jn-01 powder sample or in its composites with MCG. 

However, there are certain peaks in infrared (IR) spectra which allowed the confirmation of two polymorphs (SOD and *dia*(Zn)) in ZIF-8. As evident from the literature, ZIF-8 mainly has a 3D skeleton structure with a sodalite (SOD) topology and pore apertures of 3.4 Å in diameter [50], while ZIF-L is also related to SOD topology with a 2D layer-like structure and contains a large cavity (9.4 Å-7.0 Å-5.3 Å). The layered structures of ZIF-L are linked by N-H···N hydrogen bonds of non-coordinated HmIm species [51]. 

As shown in [46], the strongest confirmation of the existence of the SOD phase in the complex structure of ZIF-8 can be demonstrated in the IR spectrum in the region of 640–800 cm^−1^ (see Figure S5a—our data, compared with Figure S5b and Table S2 from [46]). As seen in our data in Figure S5a, the IR spectrum of jn-01 powder showed peaks at 755, 706 and 672 cm^−1^, which correlates with the literature peaks at 758, 702, and 676 cm^−1^ as shown in Table S2, and related to the SOD polymorph [42]. In addition, a peak at 1179 cm^−1^ (corresponding to 1175 cm^−1^ in Table S2 for SOD) is also shown for sample jn-01 in Figure 2a (and in Figure S5c), as well as for samples jn-04 and jn-07 (ZIF-67).

**Figure 3:**
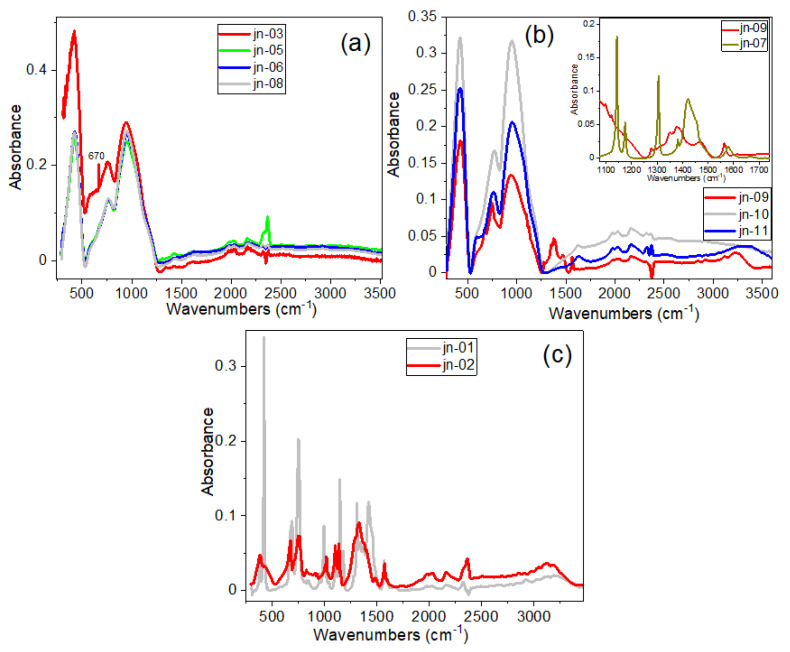
(**a**) FTIR spectra of ZIF-8/67 with MCG plates; (**b**) FTIR spectra of ZIF-67-2 + MCG_e_ (jn-09-red) in comparison with the bare MCG with (MCG_u_—blue) and without (MCG_e_—grey) HF treatment. Inset shows comparison between FTIR spectra of ZIF-67-2 + MCG_e_ and powder ZIF-67-2 in the region 1073–1742 cm^−1^ (**c**) FTIR spectrum of ZIF−8 powder sample with MCG_u_ composite sample. (Note: the subscripts “u” and “e” represent unetched and etched MCG, respectively).

The FTIR spectra of ZIFs incorporated onto microchannel glass are shown in Figure 3. We note that the FTIR spectra of composite samples after impregnation of ZIFs onto pores of the MCG plates were not very informative. This was due to the fact that, in this case, the technique used for the purpose of recording FTIR spectra (namely, with a diamond tip pressed to the surface of the sample) made it possible to obtain the spectrum mainly from the surface of the microchannel glass plate (see Figure S1a), rather than from the inside the MCG micropores. This statement is confirmed by the IR spectra of clean unetched and etched MCG (samples jn-10 and jn-11, respectively), as seen in Figure 3b. Some ZIF features in a wide spectral range were manifested only in the spectrum of sample jn-02 (ZIF-8@MCG), and in the IR spectrum of sample jn-09 for a short region of 1270–1600 cm^−1^ and in a high-frequency region of about 3250 cm^−1^ (Figure 3c), which probably occurred due to the insignificant amount of ZIFs present on the MCG surface.

Comparison of the FTIR spectrum of the ZIF-67-2 powder sample (sample jn-07), with the spectrum of its composite (sample jn-09), as shown in the inset of Figure 3b, has demonstrated that some of these features may be traces of ZIF or other moieties. There is also a rather significant band shift in the high-frequency region around 3250 cm^−1^, which can be explained as follows. ZIF-67 was incorporated into MCG after treatment (<1 min) and without treatment of MCG plates with HF. The MCG plates were treated with HF to activate or expose the functional groups of the MCG plates prior to their treatment with ZIF-67 samples. It is likely that with the rapid processing of HF, microchannel glass can expose some functional groups, such as -OH without damaging the structure. This can be seen from the IR spectrum shown by the blue line in Figure 3b, where the -OH band, which appeared at 3300 cm^−1^, shifted after treatment with ZIF-67. In the IR spectrum of the ZIF-67 composite sample with etched MCG plates (sample jn-09), peaks corresponding to MCG plates, as well as peaks corresponding to ZIF-67 (at ~670 and 749 cm^−1^) and a group of bands at ~1275, 1348, 1382, 1470 and 1563 cm^−1^), were found [46,47,48,49]. For comparison, refer to the inset of Figure 3b which has shown the IR spectrum of a pure ZIF-67 powder sample in the range 1100–1700 cm^−1^. The strong absorption bands of the MCG plate masked some of the ZIF-67 bands in this FTIR spectrum.

### 3.3. Raman Spectra

**Figure 4:**
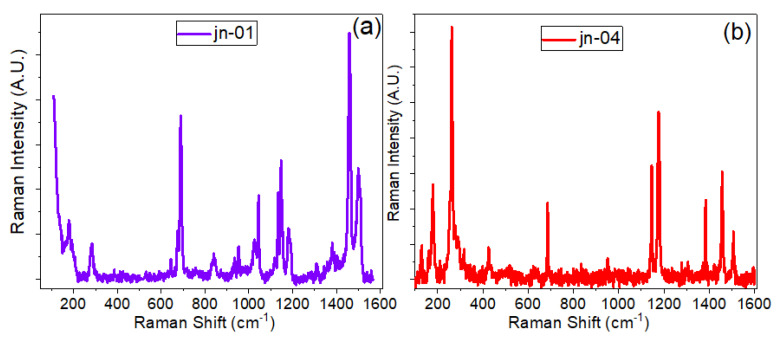
Raman spectra of (**a**) ZIF-8 and (**b**) ZIF-67-1.

The Raman spectra of powder samples jn-01 and jn-04 are presented in Figure 4a,b. In the Raman spectra, peaks are seen that match up with the peaks of the corresponding ZIFs, which is in good agreement with data from the literature [52,53,54,55,56,57,58]. As seen in Figure 4a, sample jn-01 shows the vibrational band characteristics of ZIF-8 (ZIF-L) that have been previously published in various papers [52,56]. In particular, the most intense bands are observed at 686, 1146, and 1458 cm^−1^ in the range of 500–3200 cm^−1^, which correspond to the out-of-plane bending vibration of the imidazole ring, C5–N stretching vibration, and methyl bending vibration, respectively [52,56]. In addition, the antisymmetric C-H stretching vibration in the methyl group is about 2930 cm^−1^, and the C-H stretching vibration of the imidazole ring appeared at 3131 cm^−1^. 

**Figure 5:**
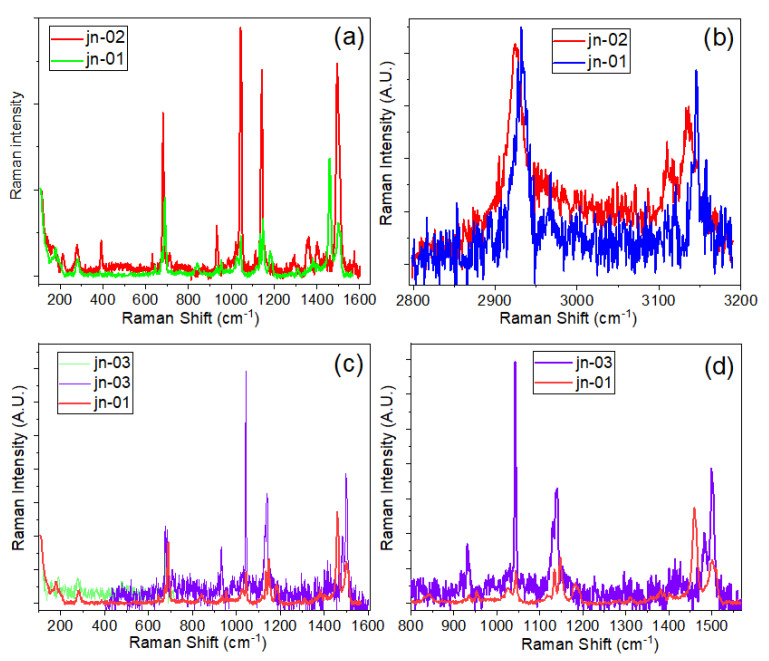
Comparison of Raman spectra of pure ZIF-L/-8 (jn-01) powder sample and ZIF-8 impregnated into (**a**,**b**) unetched (jn-02) and (**c**,**d**) etched (jn-03) pores of MCG plates shown in different regions. More detailed spectral presentation can be seen in Figure S6.

Although there are not many reports on the Raman analysis of ZIF-L and ZIF-8 structures, an analysis of the high-frequency region between 2800 cm^−1^ and 3300 cm^−1^ shows a promising result for using spectroscopic techniques to unambiguously infer the difference between the structures of ZIF-8 and ZIF-L (see [59]). As demonstrated in this report [59], one of the important differences between ZIF-8 and ZIF-L can be seen in the high frequency region, where only a band at around 3160 cm^−1^, attributed to N-H stretch, is clearly present for ZIF-L (see Figure 5 and Figure S6 from [59]). In addition to this, as also seen from Figure S6, this region, where C-H and N-H stretching bands appeared, clearly demonstrates several other differences between pure ZIF-8 and pure ZIF-L structures. In particular, the peak position of the C-H band at 2925 cm^−1^ is shifted to the low-frequency side for ZIF-L, and the relative intensity of this band with respect to the main band at 3135 cm^−1^ is completely opposite for both structures. 

At the same time, in the region of 3100–3150 cm^−1^, ZIF-8 demonstrates a perfect doublet, which is more complicated for ZIF-L in this region, accompanied by broadening of the bands and the appearance of a shoulder on the high-frequency side of the 3135 cm^−1^ band. Analysing this area for samples jn-01, jn-02, and jn-03 (see Figure 4c and Figure S6), we can conclude that sample jn-01 contains mostly ZIF-L, but inside the pores of MCG, ZIF-8 structure is mainly present. From these results, it can be concluded that the ZIF-8 structure was preserved inside the pores, but was fragile and unstable in the powder form and converted to ZIF-L structure under exposure to the ambient conditions. This is also confirmed from the previous investigations on ZIF-8 and ZIF-L where the structural changes happen not by the dissolution-recrystallisation process but rather by mere sliding of the two adjacent layers [44]. In the present investigation, it is plausible that the precise and confined regions of the MCG substrate prevented any rearrangements of the sliding layers and as such maintained the ZIF-8 structural arrangements insides the pores of MCG substrates compared with the powder samples which predominantly showed the ZIF-L morphology.

Comparison of the Raman spectra of ZIF-67 powder samples (Figure S7a–c) prepared in two different ways (with and without surfactant) indicated no differences in Raman scattering modes and its position, which confirms the structural equality in the powders.

A detailed listing of all Raman active peaks for ZIF-8, ZIF-67, and their MCG composites is presented in Table S3 and Table S4 along with the vibrational band assignments given in [60,61,62]. As seen in Table S3, some new Raman modes appeared for the composite sample compared to the powder sample. For example, the Raman spectra for ZIF-8 and its composite with MCG are shown in Figure 5a–d. Figure 5 and Figure S8 and Table S3 and Table S4 show that the appearance of new Raman modes at 213, 392, 1359 cm^−1^, and the shift of some peak positions in the Raman spectra can be attributed to the Zn-O-Si bond and, therefore, to the interaction of ZIF-8 with the MCG matrix. Since microchannel glasses consist predominantly of silicates [63], the interaction of the oxygenic functional groups (from MCG) and metal groups in ZIF leads to the anchoring of ZIF on the structure of microchannel glasses. Other peaks are related to stretching and bending of the imidazole rings, the main ones being at ~279, 681, 1142, 1494, and 1505 cm^−1^, which may be due to Zn-N stretching, imidazole ring puckering, C5-N vibrations, methyl bending, and C4=C5 stretching, respectively, similar to those described in the literature [56].

Despite less noticeable changes for different polymorphs in the Raman spectrum for complex ZIFs, the Raman spectra of the powder sample jn-01 also confirmed the presence of the SOD structure, as shown in the [42] (see Figure S9 and Table S5), as well as in our spectra with the appearance of the 935 cm^−1^ band (together with the 953 cm^−1^ band) for the jn-01 powder sample. Table S5 [42] shows the presence of the SOD phase, in this case, can also be confirmed by the simultaneous absence of the band at 759 cm^−1^. It should be noted that the Raman spectra, recorded for composites based on jn-01 powder and MCG plates (samples jn-02 and jn-03), demonstrated the presence of a peak at 933 cm^−1^ and a complete absence of the band at 759 cm^−1^ (see also Figure 7a,b below). From our point of view, this enables us to conclude that a stable ZIF structure with a predominance of the SOD phase is formed inside the pores of the MCG plates. This provides the advantage of using the micro-Raman technique to detect SOD/*dia*(Zn) polymorphs infiltrated into micron-sized porous structures. 

**Figure 6:**
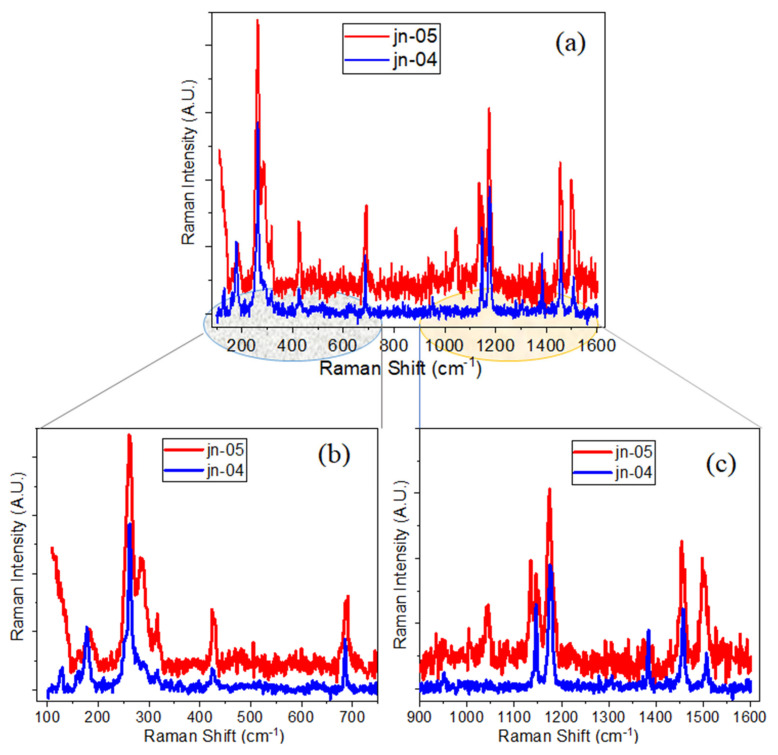
Raman spectra of (**a**) ZIF-67-1 powder sample (blue) and ZIF-67-1@MCG (red); (**b**,**c**)—show the more detailed spectra in lower and higher frequency ranges.

A comparison of the Raman spectra of the ZIF-67-1 (jn-04) powder sample with ZIF-67-1@MCG composite samples (jn-05 and jn-06) is presented in Figure 6 and Figure S10. Raman spectra of jn-04 and jn-05 essentially show the same peak positions with varying intensities. However, in the region of higher frequencies, the appearance of a new Raman peak at ~1044 cm^−1^ can be seen for the composite sample, which can be attributed to the bridging oxygen Si–O–metal (Co) from ZIF-67. This is also visible for the ZIF-67-2 composite sample with etched MCG (see Figure S10). Similar to that of ZIF-8 composite samples, new Raman modes can be seen for these composite samples (for example, at ~286 cm^−1^), which can be related to the vibration frequencies of the newly formed bonds between ZIF-67 and the MCG plates. 

**Figure 7:**
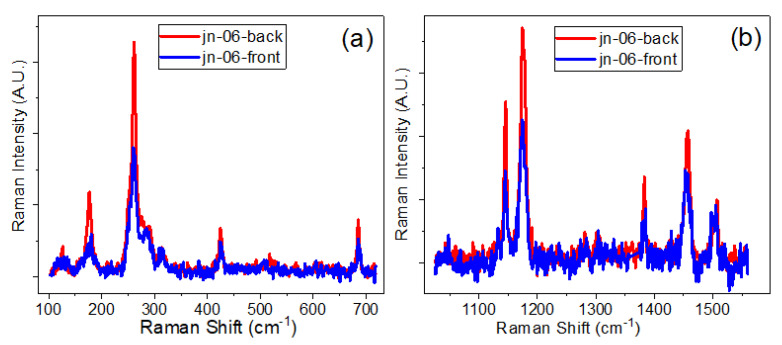
Raman spectra measured in different regions, (**a**) 100–750 cm^−1^ and (**b**) 1000–1600 cm^−1^, for ZIF-67-1 infiltrated into etched MCG channels, sample jn06, from the front (blue line) and from the back (red line) sides of the MCG plate.

To check the degree of infiltration of MCG channels by ZIFs, Raman spectra were recorded inside pores from the front and back sides of MCG plates. Examples of the Raman spectra measured in this way for samples jn-05 and jn-06 (ZIF-67-1 in MCG_u_ and MCG_e_, respectively) are shown in Figure 7 (sample jn-06) and Figure S11 (sample jn-05). As can be seen from these figures, the spectra recorded on both sides of the MCG plates are almost identical. This confirms the either partial or complete infiltration of MCG channels with ZIF compounds. In addition, optical images (Figure S1) from the Raman microscope confirm uniform filling of ZIFs inside the MCG substrates.

**Figure 8:**
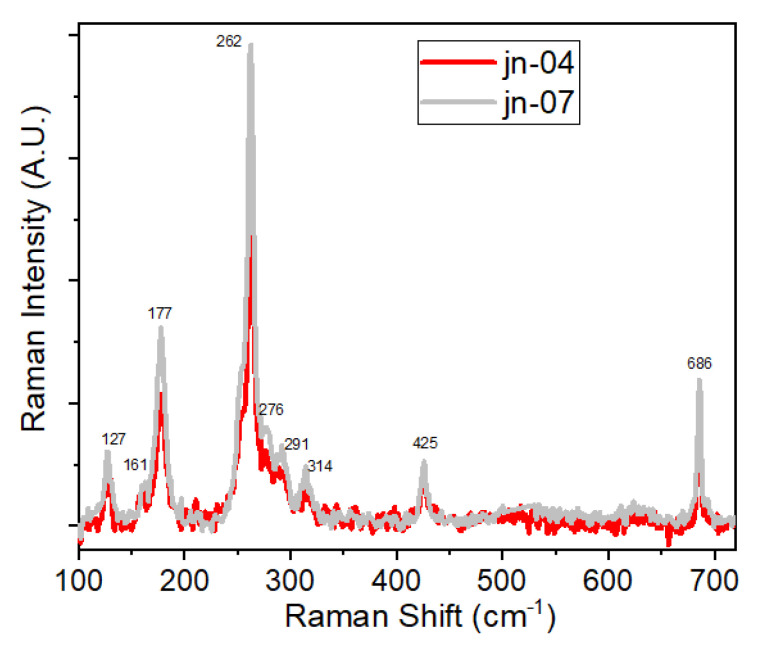
Raman spectra of ZIF-67-1 and ZIF-67-2 powder samples shown in the region 100–750 cm^−1^ to demonstrate the low-frequency peaks at 121, 161, and 177 cm^−1^.

Finally, it should be noted that in some cases (see Figure 8 and Table S3) in the low-frequency part of Raman spectra, a vibrational band at ~127 cm^−1^ was also seen. For the powder form it was mainly seen as a case of absence of a strong background signal in this region, which was the case when powder was dissolved in a neutral solution of micropore water, followed by a long sonication and finally casting onto piece of Si wafer. It was also registered for some ZIF + MCG composites (see Table S3 and Figure 6b and Figure S10a). Low-frequency part of vibrational spectra (viz low-frequency Raman and far-infrared or THz absorption) recently attracted a great deal of attention from researchers due to the importance of this region and to draw conclusions based on low-energy collective modes (or lattice vibrations) [64,65,66,67]. This is precisely where relations between the physical properties and the lattice dynamics can be investigated, since soft modes (e.g., breathing modes of the framework), gate-opening, and shearing are linked to adsorption, elasticity, structural transition, and instability [65,66,67].

As demonstrated in a number of recent theoretical and experimental works [64,65,66,67,68,69,70], in the region of 170–280 cm^−1^ bending and stretching modes of Zn-N units, in the case of ZIF-8 and Co-N units for ZIF-67, were observed, and this can resulted in distortion of the linker (imidazolate) unit and small structural deformations of ZIFs inside the pores. In the region below 150 cm^−1^ some important physical phenomena prevail, such as gate-opening, pore breathing, shearing, as well as other structural mechanisms responsible for the fundamental properties of the framework. 

As far as the above-mentioned band at 127 cm^−1^ is concerned, in accordance with [69], it is assigned to the asymmetric gate opening (+methyl rotation) and is active in both IR and Raman spectra. This was confirmed in theoretical calculations (using DFT and ab initio methods) as well and experimentally using synchrotron THz and Raman measurements as per the investigations reported for ZIFs [63,64,65,66,67,68,69] including ZIF-8 and ZIF-67. 

With regard to the mechanism of growth of ZIF on MCG, it is clear from spectroscopic studies, as outlined above, that the silanol groups of MCG play a role in the condensation reaction between ZIF and MCG substrates. It is well known in the literature that condensation can occur between the silanol group or hydroxyl groups of nanomaterials, and in this case it is caused by the high temperature created when using a Teflon-coated autoclave for this purpose. However, further studies will focus more on the morphology of the ZIF materials that form inside the MCG, which may provide more information about the type of ZIF materials (microplates, thin nanosheets, or rhombic dodecahedron morphology) since solvent molecules can alter the morphology of ZIF materials [71] and can be used to determine the ZIF orientation.

## 4. Conclusions

In this work, we demonstrated a fabrication technique for ZIF growth on microchannel glass substrates. The XRD and spectroscopical studies suggest the formation of predominantly ZIF-L phase in the powder form, whereas ZIF-8 is within the pores of MCG. The XRD and spectroscopy data confirms the presence of two phases (SOD and *dia*(Zn)) with a higher content of the SOD phase in the composite samples. FTIR and Raman studies of the samples have revealed the fundamental aspects of the bonding in ZIFs and ZIFs@MCG composites. Despite the moisture-sensitive nature of MOFs and ZIFs, the powders reported in our studies were intact inside the porous substrate, demonstrating the advantage of ZIFs@MCG composites. Moreover, our method has the advantage of not requiring seed materials to promote the growth of ZIFs on a porous substrate. However, in future studies, investigations will be focused on how the ZIF structures can be modified with the use of different substrates and solvents. As the results have demonstrated, ZIF powders in microchannel glass can provide stable ZIF structures in pristine conditions, protected from an oxidizing atmosphere or moisture, which is important for the stability of organometallic framework structures. It has also been demonstrated that micro-Raman spectroscopy can be used as a built-in characterization tool for evaluating the mass production of ZIF structures inside pores, as well as molecular guest particles during various chemical reactions. We believe that these composite materials will have a wide range of important applications in the future.

## Data Availability

Not applicable.

## Supplementary Materials

The following supporting information is available online at https://www.mdpi.com/article/10.3390/ma16062410/s1, Figure S1(a). White light image of sample JN-02 (ZIF−8 impregnated into MCG (microchannel glass) pores. Figure S1(b). White light images of sample JN-05 (ZIF-67-1) impregnated into unetched MCG pores. Figure S1(c). White light images of sample JN-08 (ZIF-67-1) impregnated into unetched MCG pores, and of sample JN-09 (ZIF-67-2), impregnated into etched MCG pores. Figure S2. XRD patterns for (a) original sample jn-01 (corresponding to XRD patterns for ZIF-L) and (b) the same sample after heating at 65 °C for 150 hours (corresponding to XRD patterns for ZIF−8). Figure S3. SEM images of samples (a) jn-02 (ZIF-L/-8)@MCG) and (b) jn-06 (ZIF-67@MCG). Figure S4. FTIR spectra of ZIF-67 prepared using two different routes as outlined in the experimental section. Figure S5(a) FTIR spectra of powder samples jn-01 and jn-07 and compared it will the Figure S5(b) taken from (Reference [1] in SM). Figure S5(c) demonstrates the presence of the band at 1179 cm^−1^. Figure S6. Taken from Reference [2] in SM to show the Raman spectra of crystals obtained under various conditions. Figure S7. Raman spectra of ZIF-67-1 prepared using two different routes of preparation as outlined in the experimental section (a) in the frequency range 100–1600 cm^−1^, (b) and (c) show more detailed comparison at low and high frequencies, respectively. Figure S8. Comparison of Raman spectra of pure ZIF-8 (jn-01) powder sample and ZIF-8 impregnated into unetched (jn-02) pores of MCG plates shown in detail in different spectral regions (a to d). Figure S9. (a) Raman spectra of powder sample jn-01 (ZIF-8), shown in the region 700–1120 cm^−1^ and (b) of composite samples jn-02 and jn-03 in the region of 580–1230 cm^−1^. Figure S10. Raman spectra of composite samples with ZIF-67-1 (jn-04) impregnated into unetched MCG (jn-05-red line) and into etched MCG (jn-06-blue line) shown in regions (a) 90–720 cm^−1^ and (b) 1600–1630 cm^−1^. (c) Raman spectra for powder samples ZIF-67-2 (jn-07) and its composite with etched MCG plate (jn-09). Figure S11. Raman spectra measured in different regions, (a) 100–750 cm^−1^ and (b) 1000–1600 cm^−1^, for ZIF-67-1 infiltrated into unetched MCG channels for sample jn05 from the front (blue line) and from the back (red line) sides of the MCG plate. Table S1. Samples description. Table S2. IR band assignment of ZIF phases (taken from Reference [1] in SM). Table S3: Comparison of Raman data for all studied samples. Table S4. Wavenumbers (υ) and band assignments from review of literature, shown in square brackets. Table S5. Raman band assignments of ZIF phases (taken from Reference [1] in SM). SOD polymorph related bands are denoted with *. Reference [72] are cited in the supplementary materials.
