# Supplementary material for: XRD and Spectroscopic Investigations of ZIF—Microchannel Glass Plates Composites
Source: Materials (Basel). 2023 Mar 17;16(6):2410. doi: 10.3390/ma16062410 (PMC10056852; doi:10.3390/ma16062410)
Supplement: Supplementary file 1 [file materials-16-02410-s001.zip › materials-2232391-supplementary.pdf]

Supplementary Materials

XRD and Spectroscopic Investigations of ZIF−Microchannel Glass Plates Composites

Justin Narimbi[1], Sivakumar Balakrishnan[1], Tatiana S. Perova[2], Garret Dee[3], Gerhard F. Swiegers[4], Yurii K. Gun'ko[3]

[1]Department of Applied Sciences, The PNG University of Technology, Lae, Morobe Province, MP 411, Papua New Guinea; justin.narimbi@pnguot.ac.pg (JN); sivakumar.balakrishnan@pnguot.ac.pg (SB)
[2]Department of Electronic and Electrical Engineering, Trinity College Dublin, The University of Dublin, D02 PN40, Ireland; perovat@tcd.ie (TSP)

[3]School of Chemistry, Trinity College Dublin, The University of Dublin, D02 PN40, Ireland; deeg@tcd.ie (GD); igounko@tcd.ie (YKG)

[4]Intelligent Polymer Research Institute, University of Wollongong, NSW 2522, Australia; swiegers@uow.edu.au (GFS)

Table of Contents

Contents **Page**

I. Samples description (Table S1) 2
II. White-light images of composite samples ZIFs+MCG plate (Figure S1) 3
III. XRD data (Figure S2a,b) 4
IV. Interpretation of EDX and SEM data (Figure S3a,b) 5
IV. FTIR data (Figure S4, S5)
6
V. FTIR: discussion of two phases in ZIF-8 (Table S2) 7
VI. Raman data (Figure S6, Figure S7)
7–8
(Table S3, Table S4) 9–10
VII. Raman data of powder and composite samples (jn-02 vs jn-01) (Figure S8) 11
VIII. Raman data for two phases in XIF-8 (Figure S9, Table S5) 12
IX. Raman data for ZIF-67-1 and ZIF-67-2 (Figure S10) 13
XI. Raman data registered from both sides of MCG plates (Figure S11) 13
References: 14

I. Samples Description

Table S1. Samples description

Sample Code	Type of ZIFs	Sample Description
JN-01	ZIF-F/-8 (powder sample)	The ZIF-8 crystals were dried at room temperature after washing with methanol.
JN-02	ZIF-L/-8/MCG_u (unetched)	ZIF-8 with microchannel glass dipped in before heating at 150 °C .
JN-03	ZIF-L/-8/MCG_e (etched)	Same as JN-02 except in this case an etched microchannel glass was used instead of an unetched microchannel glass.
JN-04	ZIF-67-1 (powder)	ZIF-67 crystals dried in an oven.
JN-05	ZIF-67-1/MCG_u (unetched)	ZIF-67 with microchannel glass (suspended in ZIF-67 mother solution).
JN-06	ZIF-67-1/MCG_e (etched)	Same as JN-05, except the microchannel glass was etched.
JN-07	ZIF-67-2 powder	A different procedure for ZIF-67 synthesis (given under separate heading as “ZIF-67 route two”)
JN-08	ZIF-67-2/MCG_u (unetched)	Same solution as JN-07. In this case unetched microchannel glass was used (suspended in ZIF-67 mother solution) in “route two method” ZIF-67.
JN-09	ZIF-67-2/MCG_e (etched)	Same procedure as JN-08 except that an etched MCG was used.
JN-10	MCG_u (original, unetched) alone	MCG alone sample without any treatment.
JN-11	MCG_e (etched)	MCG alone sample with chemical treatment

II. White light images of composite samples ZIFs + MCG plate

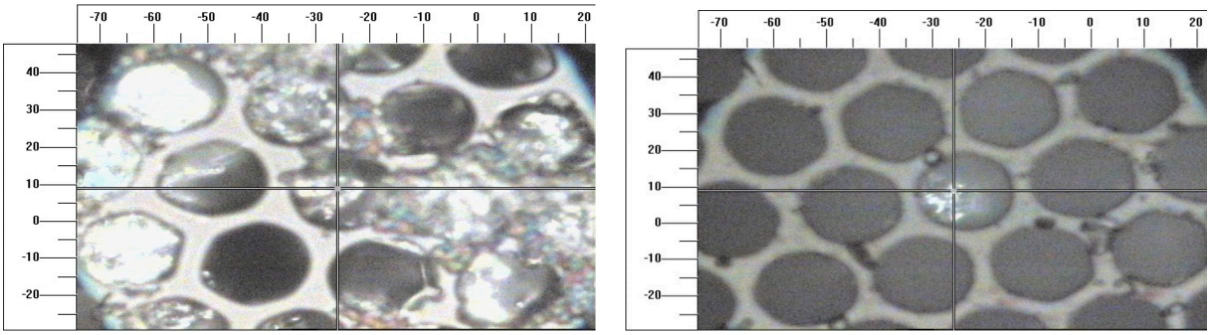

Figure S1(a). White light image of sample JN-02 (ZIF-8 impregnated into MCG (microchannel glass) pores

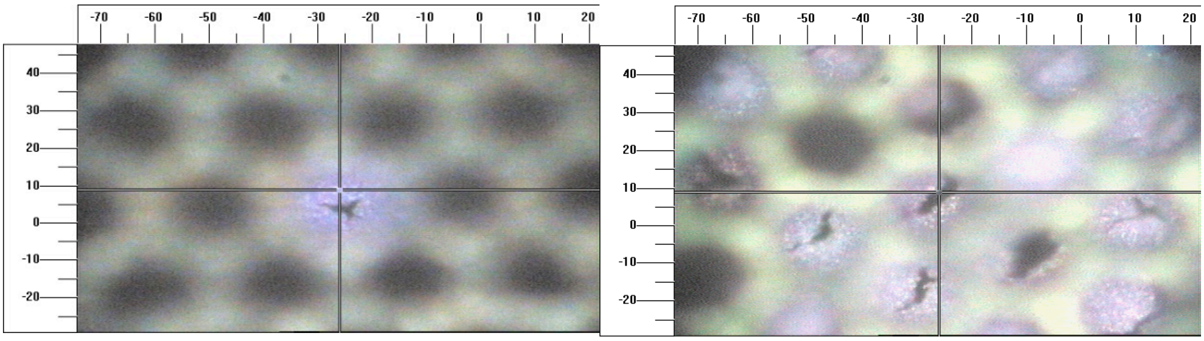

Figure S1(b). White light images of sample JN-05 (ZIF-67-1) impregnated into unetched MCG pores.

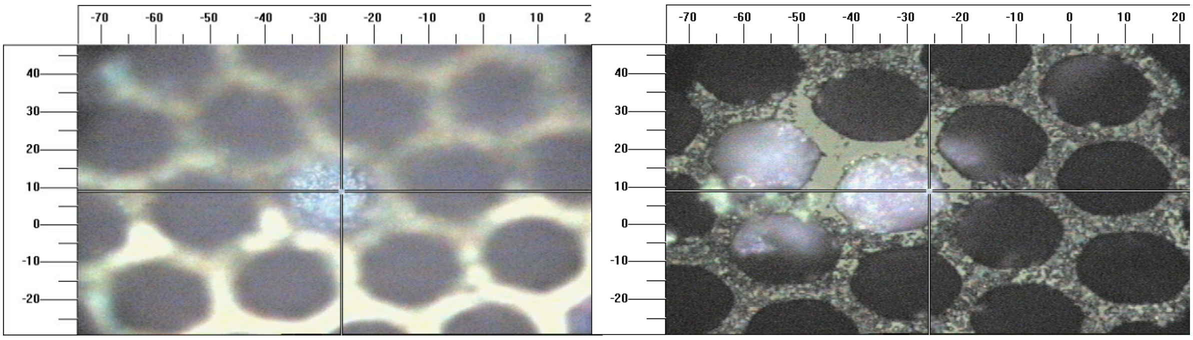

Figure S1(c). White light images of sample JN-08 (ZIF-67-1) impregnated into unetched MCG pores—left panel, and of sample JN-09 (ZIF-67-2)—right panel, impregnated into etched MCG pores.

III. XRD data

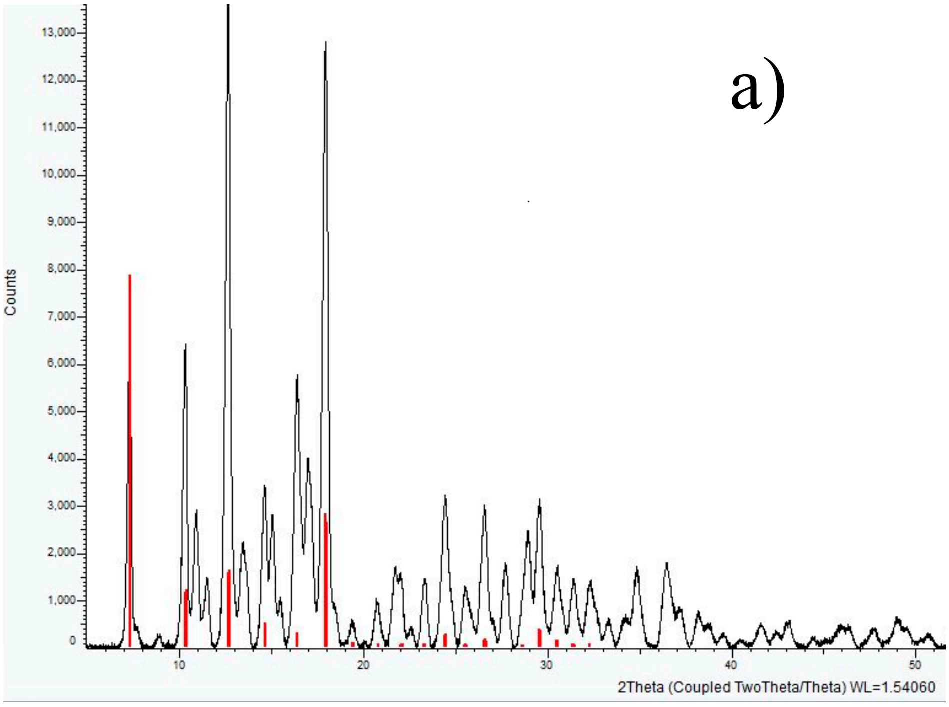

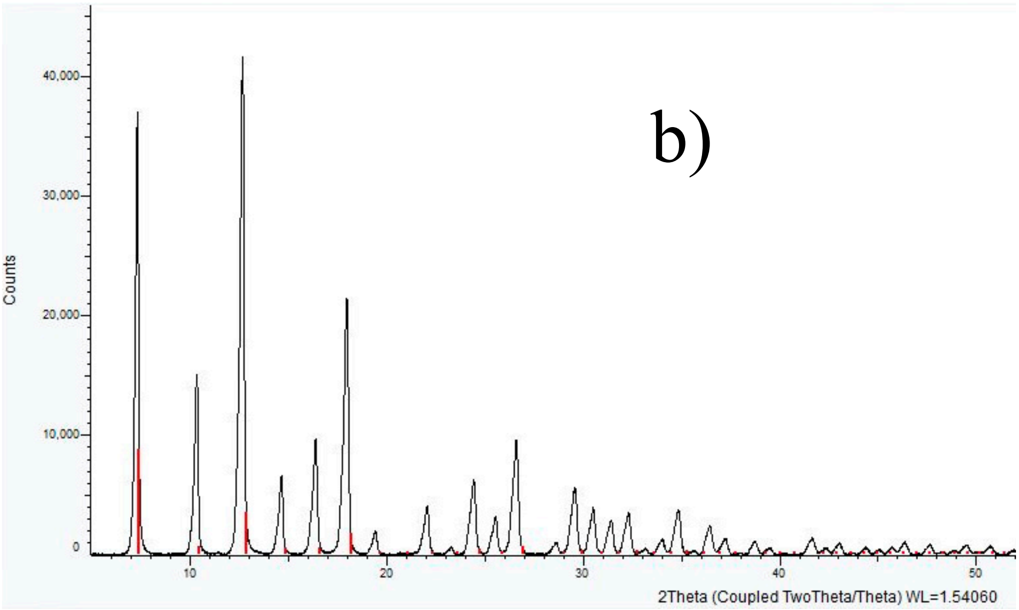

Figure S2. XRD patterns for (a) original sample jn-01 (corresponding to XRD patterns for ZIF-L) and (b) the same sample after heating at 65 ºC for 150 hours (corresponding to XRD patterns for ZIF-8). The XRD results clearly demonstrated a change in the crystal structure, but the bulk morphology had not changed significantly as observed by the SEM data below. This indicates that the material is a polymorph, a mineral changed on the molecular level only, where the transformed mineral looks identical to the original unaltered form.

IV. EDX and SEM data

From the EDX measurements results it is evident that the material that has grown inside the pores of MCG are ZIF-L/-8 and ZIF-67. The EDX shows the presence of Zn (for ZIF-L/-8) and other elemental peaks corresponding to the sample holders that we have used for analysis. Similarly, for the ZIF-67, the presence of Co is confirmed along with the peaks attributed to the sample holder and TEM grid.

The SEM image of the ZIF-67 powders (not shown here) shows a polyhedral morphology with a particle size of around 300 nm, which demonstrates the successful synthesis of ZIF-67.

The SEM images of (ZIF-L/-8)@MCG, ZIF-67@MCG show more or less uniform filling of (ZIF-L/-8) and ZIF-67 inside the pores of MCG. Although the SEM image has shown that some pores were not filled completely, it is evident that ZIF growth has happened and it was filling at different depths. The shape of the ZIF crystals can vary and it depends strongly on the reaction conditions. In the case of ZIF-8@MCG, the ZIFs inside the pores could be highly intergrown with no clear distinction of leaf-shaped morphology (as we would expect for ZIF-L) or the polyhedral morphology as that of ZIF-8. This conclusion supports the observations we made with Raman spectroscopy.

a) b)

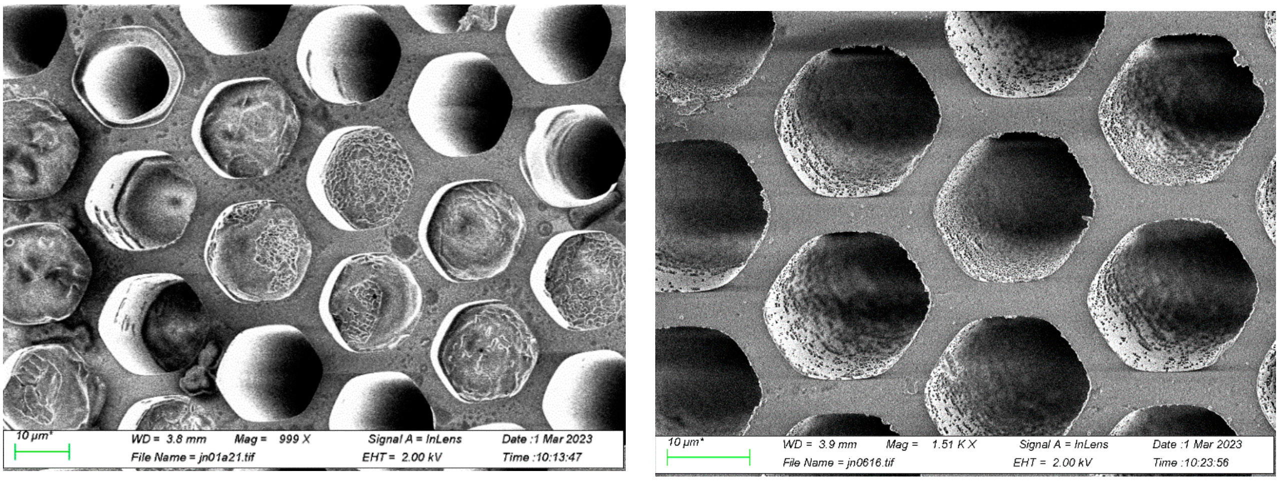

Figure S3. SEM images of samples (a) jn-02 (ZIF-L/-8)@MCG) and (b) jn-06 (ZIF-67@MCG).

Although, the current study was focused on the Raman and FTIR studies on ZIFs@MCG, future study will focus on SEM analysis of cross-section which will enable to find out the depth to which the ZIFs materials have filled.

V. FTIR data

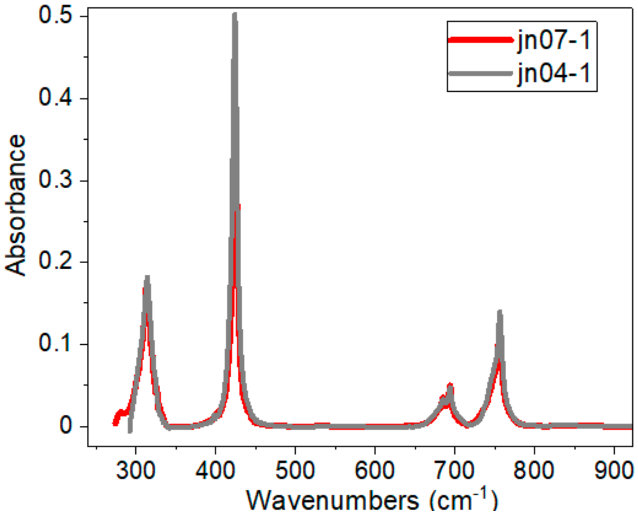

Figure S4. FTIR spectra of ZIF-67 prepared using two different routes as outlined in the experimental section.

VI. FTIR data discussion for two phases in ZIF-8 studied in this paper.

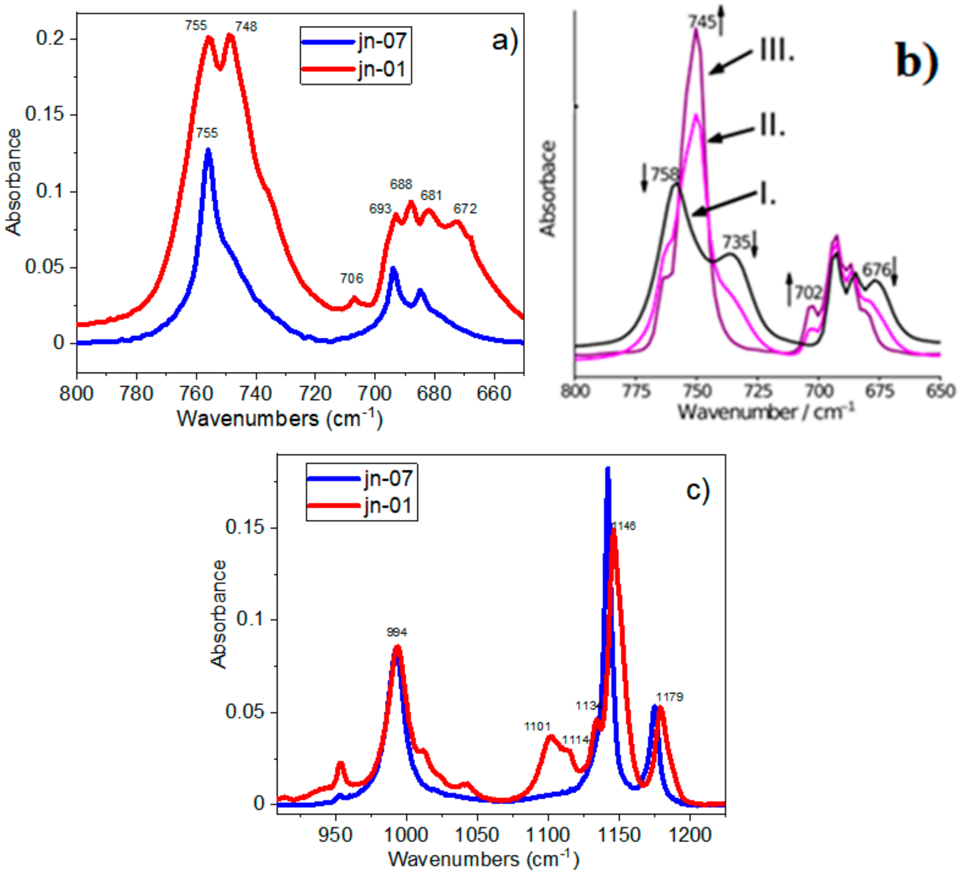

Figure S5. a) FTIR spectra of powder samples jn-01 and jn-07 in the region where the mixture of two phases for ZIF-8 (jn-01) was the most pronounced. This can be confirmed by Figure b) taken from [1] as well as by Table S2 which lists the vibrational bands assignment. Figure c) demonstrates the presence of the band at 1179 cm^{-1} which is close to 1175 cm^{-1} in Table S2.

Table S2. [1] IR band assignment of ZIF phases. SOD polymorph related band are denoted with *.

Wavenumber / cm^{-1}	Band assignment
421	Zn–N stretching
676*<br>692<br>702*<br>745 / 758*	Imidazolate ring puckering,<br>H out of plane bending
996<br>1143<br>1168 / 1175*<br>1307	Imidazolate ring puckering,<br>H in plane bending
1418<br>1460	Imidazolate ring stretching

VII. Raman data

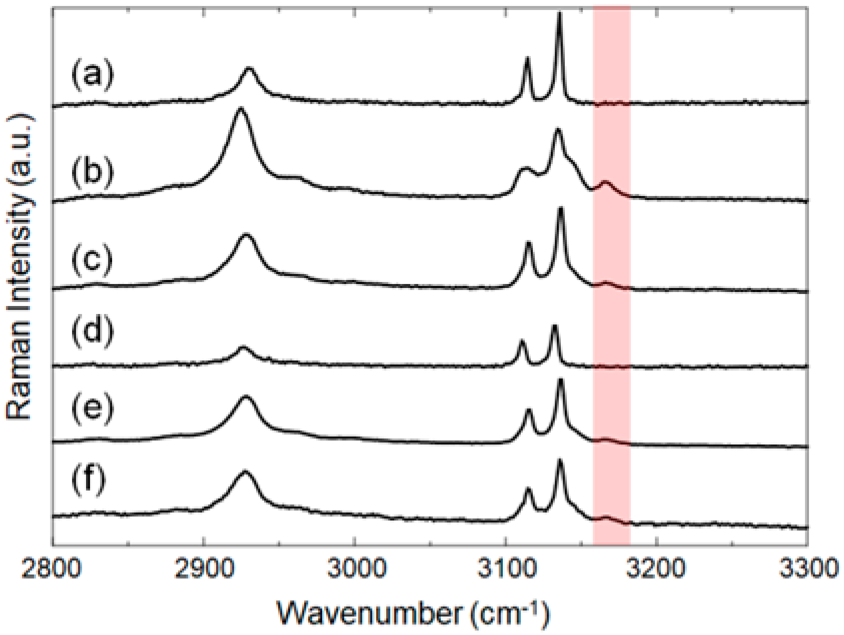

Figure S6. (From [2]) Raman spectra of crystals obtained under various conditions: (A) reference sample of ZIF-8, (B) reference sample of ZIF-L, (C) ZIF crystals formed in free

aqueous solution (no optical cavity), (D) ZIF crystals formed in an optical cavity where the water OH stretch is strongly coupled to vacuum fields, (E) ZIF crystals formed on single mirror, and (F) ZIF crystals formed in cavity cell without Au mirrors sputtered on the ZnSe windows (i.e., a confined space but not an optical cavity). The red shaded area indicates the energy of the N–H related stretching vibration, N–H bonding is only present in the ZIF-L.

Raman data

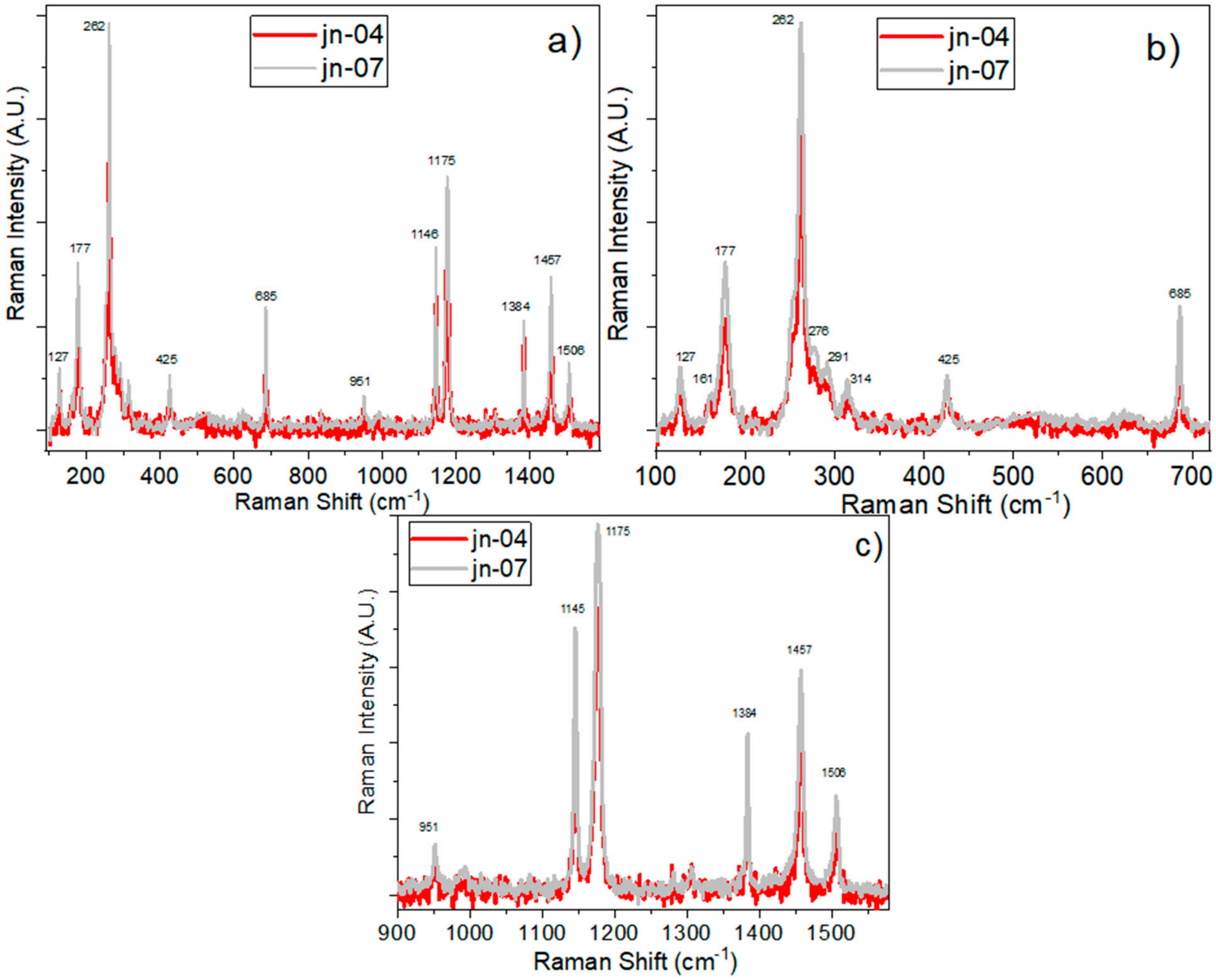

Figure S7. Raman spectra of ZIF-67-1 prepared using two different routes of preparation as outlined in the experimental section (a) in the frequency range 100–1600 cm^{-1}, (b) and (c) show more detailed comparison at low and high frequencies, respectively.

Table S3: Comparison of Raman data for all studied samples

Other data, experiment, calculations		ZIF-8 (jn-01)			ZIF-67 -1 (jn-04)			ZIF-67-2 (jn-07)		
ZIF-8 [3]	2-MIM* [4-7]	Powd. JN-01	MCGu[a] JN-02	MCGe[и] JN-03	Powd. JN-04	MCGu[a] JN-05	MCGe[и] JN-06	Powd. JN-07	MCGu[a] JN-08	MCGe[и] JN-09
					127		127	127		
168		147		159	161	163				
	176exp	179			176	182	176	177	178	180
			213	190						
					254		251	252	254	251
					262		261	262	262	261
273	272	282	279	278	276	286	277	277	283	285
	367		392				290	292		316
	421exp				316	316	314	314	315	358
	465exp				425	426	424	426	425	425
	628	644w	631							
686<br>755	678	675<br>689	681	676<br>685	685	687	685	685	687	687
833	856	841	711	709				693		
				917						
950	928<br>975	935<br>953	933	933	951			951		
1021	996	1024<br>1044	1022<br>1041					-	1043vw	
	1123	1117	1046	1043		1044			1132	1045
1146	1153	1123		1130		1134		1145	1146	1136
1180	1282	1134<br>1147	1142	1139	1146	1146<br>1151	1145	1176		1144<br>1148
1187	1351	1179<br>1191			1177	1175	1174		1173	1174
1311	1361	1310<br>1380w	1291<br>1359		1384	1184<br>1386	1179sh<br>1382		1182<br>1359	1183<br>1377
1384	1398	1402w	1401	1470	1458	1455		1384	1384	1404
1458	1477-<br>1486	1459<br>1464sh	1494	1483		1461	1457<br>1499sh	1457	1455	1455
1499	1492	1500<br>1510	1505	1501	1500<br>1507	1501<br>1518	1507	1506	1501	1501
1508	1564		1575							
2915	2882									
2931	2936	2924	2933		2928	2926	2929	2927		2923
	2959		2966	-						
	2979			-						
	3007									
	3042									

3110	3114	3113	3119	-		3114	3114	3115	3115	3114
3131	3128	3135	3141	-	3135	3135	3135	3135	3137	3136
	3156	3167								

* 2-methylimidazole (MIM), data in this column are taken from [5], see also [4,6] and [7] with recent experimental data shown in red font.

Table S4. Wavenumbers (ν) and band assignments from review of literature, shown in square brackets.

ZIF-8 [8]		ZIF-8 [3]		2-methylimidazole [4] Co-Imdz	
ν	Assignment*	ν	Assignment	ν	Assignment
		168	ν Zn–N	176exp	Co(Zn)-N) [6]
282	ν Zn-N	273	ν Zn–N	272	γ(CCH_3)
				367	δ (CCH_3)
421	exp [6]		ν Zn–N	421exp	ν Co–N [6]
				465exp	Co-O-Co [6]
642	τ Imdz ring			628	γ(R)
685	Imdz ring puckering, δ H (out of plane)	686	Imdz ring puckering, H oop bend	678	ν(CCH_3)
752	δ C=N (out of plane), δ N-H	755	C=N oop bend, δ N–H	856	γ (CH)
836	δ C-H(out of plane) (C4-C5)	833	C–H oop bend (C4–C5)	928<br>975	γ (NH)<br>δ (R)
953	δ C-H (out of plane) (C2-H)	950	C–H oop bend (C2–H)	996	δ (R), δ (CH)
1022	δ C-H (out of plane)	1021	C–H oop bend	1123	δ(CH)
1146	ν C5-N	1146	ν C5–N	1153	ρ(CH_3)
1180	ν C-N, N-H wag	1180	ν C–N + N–H wag	1282	δ(NH)
1187	ν C-N	1187	ν C–N	1351	ν(R)
1311	Ring expansion, N-H wag	1311	ring expansion + N–H wag	1361	ν(R)
1384	δ CH_3	1384	δ CH3	1398	ν(R), $δ_{sym}$ (CCH_3)
1460	δ C-H	1458	C–H wag	1477-1486	$δ_{asym}$(CH_3), sh
1499	ν C2-N1, ν C5-N1, N-H wag	1499	C2N3 + C4N3 + ν C5N1 + N-Hwag	1492	ν(R)
1510	ν C4=C5	1508	ν C4=C5	1564	ρ(CH_3) ½c(R)
		2915	$ν_{sym}$ C–H (methyl)	2882	2ν(R)
2924	ν C-H (methyl)	2931	$ν_{asym}$ C–H (methyl)	2936	$ν_{sym}$(CH_3)
3108	ν C-H (Im ring)	3110	ν C–H (ar)	2959	$ν_{sym}(CH_3)_{FR}$
3129	ν C-H (Im ring)	3131	ν C–H (ar)	2979	$ν_{asym}$(CH_3)
				3007	$ν_{asym}$(CH_3)
				3042	Overtone

				3114	ν(CH)?
				3128	ν_{asym}(HCCH)
				3156	ν_{sym}(HCCH)

*ν: stretching, τ: torsion, δ: bending, oop: out of plane, ar: aromatic, sym: symmetric, asym: asymmetric.

VIII. Raman data for comparison of powder and composite samples (jn-02 vs. jn-01)

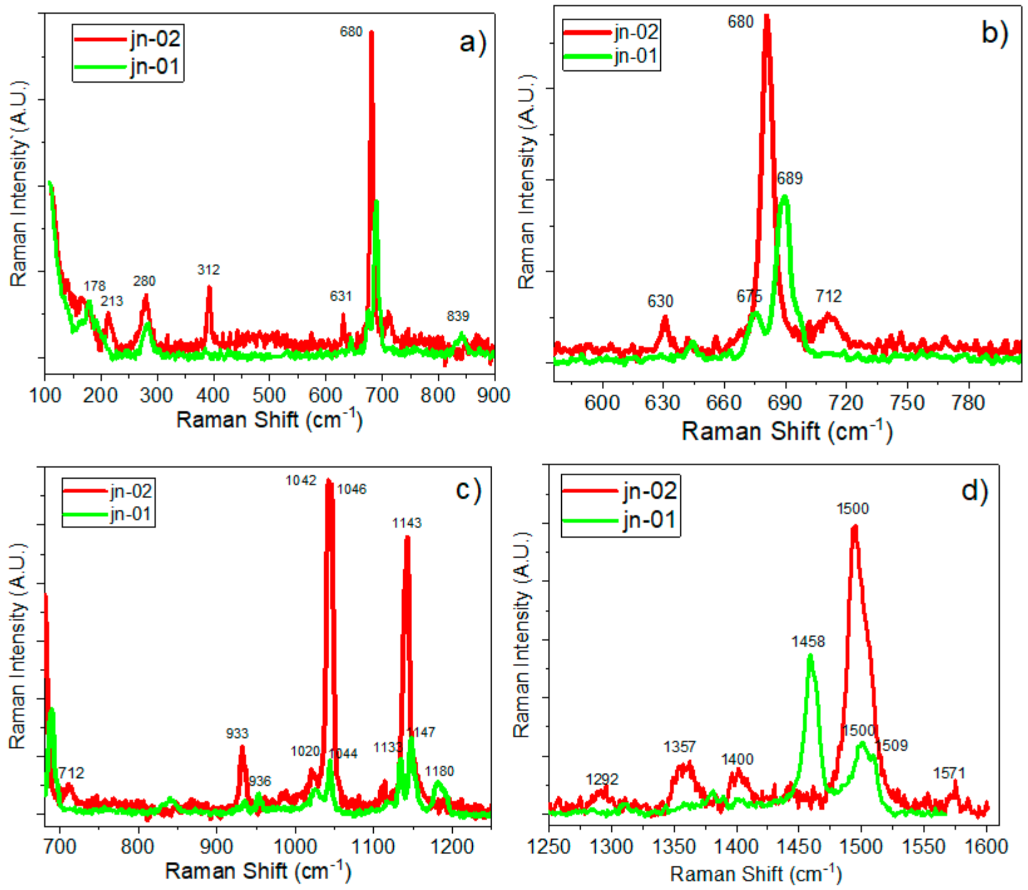

Figure S8. Comparison of Raman spectra of pure ZIF-8 (jn-01) powder sample and ZIF-8 impregnated into unetched (jn-02) pores of MCG plates shown in detail in different spectral regions (a – d). (a) spectrum from 100 to 900 cm^{-1}. (b) spectrum from 570 to 820 cm^{-1}; (c) spectrum from 700 to 1250 cm^{-1} and (d) spectrum from 1250 to 1600 cm^{-1}.

IX. Raman data for discussion of two phases in ZIF-8 powder and composites:

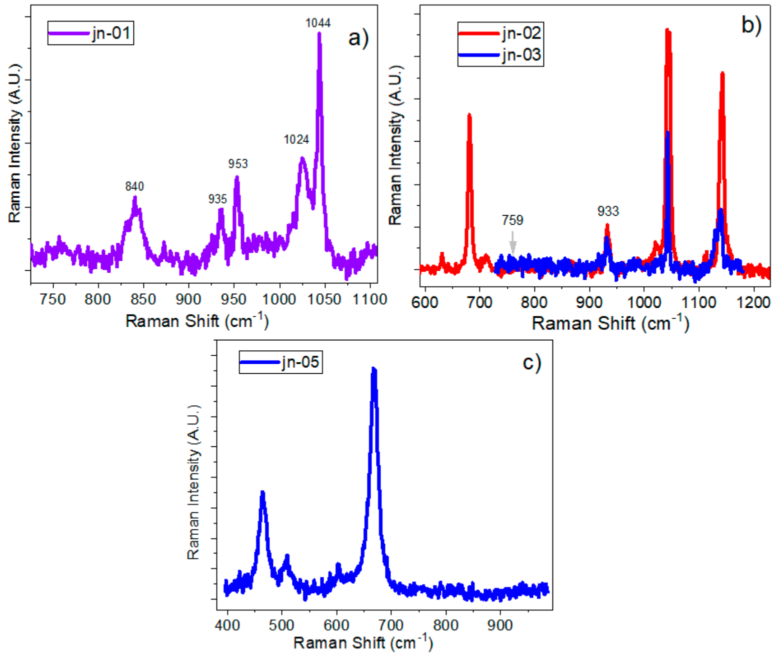

Figure S9. a) Raman spectra of powder sample jn-01 (ZIF-8), shown in the region 700–1120 cm^{-1} and b) of composite samples jn-02 and jn-03 in the region of 580–1230 cm^{-1}, demonstrating the absence of a band at 759 cm^{-1} and presence of a band at 933 cm^{-1}. This led to the conclusion that the structure of ZIF-8 formed inside the micropores of MCG plates have a substantial contribution from the SOD phase. c) Raman spectrum of the composite sample jn-05 (with ZIF-67-1 infiltrated inside the pores of the MCG plate) shown for comparison and demonstrating the absence of a band at 933 cm^{-1} in this case as well. The same is true for the rest of composite samples based on ZIF-67-1 and ZIF-67-2 such as jn-06, jn-08 and jn-09.

Table S5. Raman band assignments of ZIF phases [1]. SOD polymorph related bands are denoted with *.

Raman shift / cm^{-1}	Band assignment
645	Imidazolate ring puckering
686	Imidazolate ring puckering, H out of plane bending
759, –*	C=N out of plane bending, N–H bending
833	C–H out of plane bending (C4–C5)
945, 933*	C–H out of plane bending (C2–H)
1019	C–H out of plane bending
1143	C5–N stretching
1180	C–N stretching and N–H wagging
1311	ring expansion and N–H wagging
1378	CH_3 bending
1454	C–H wagging
1500	C2N3 and C4N3, C5N1 stretching, and N–H wagging

X. Raman data for ZIF-67-1 and ZIF-67-2 and the composites based on them

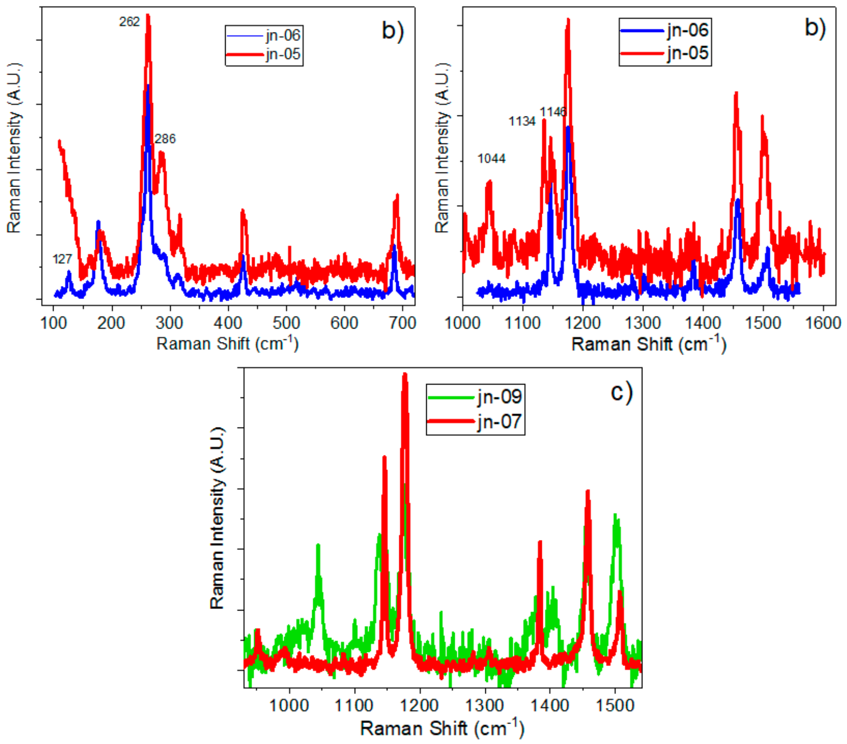

Figure S10. Raman spectra of composite samples with ZIF-67-1 (jn-04) impregnated into unetched MCG (jn-05—red line) and into etched MCG (jn-06—blue line) shown in regions a) 90–720 cm^{-1} and b) 1600–1630 cm^{-1}. c) Raman spectra for powder samples ZIF-67-2 (jn-07) and its composite with etched MCG plate (jn-09).

X. Raman spectra registered from the front and back sides of MCG plates

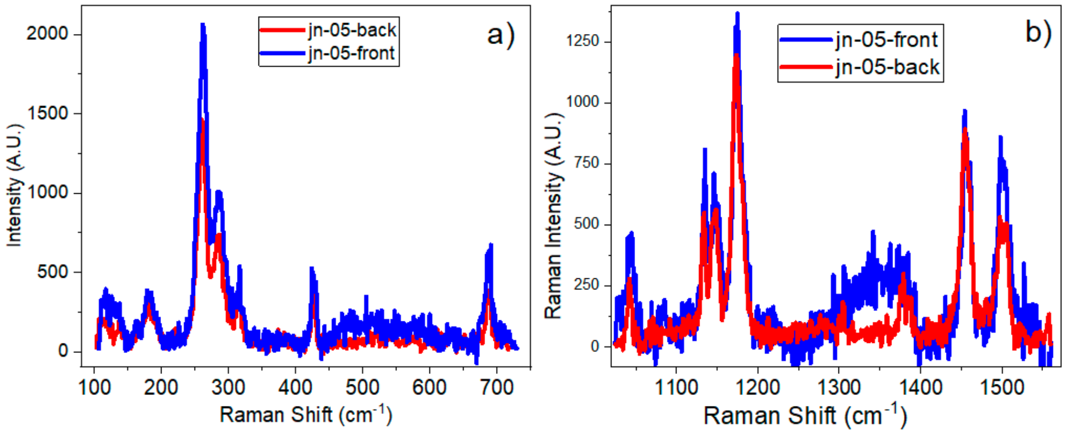

Figures S11. Raman spectra measured in different regions, (a) 100–750 cm^{-1} and b) 1000–1600 cm^{-1}, for ZIF-67-1 infiltrated into unetched MCG channels for sample jn05 from the front (blue line) and from the back (red line) sides of the MCG plate.

References:

1. Edina Balog, Gábor Varga, Ákos Kukovecz, Ágota Tóth, Dezső Horváth, István Lagzi, and Gábor Schuszter. Polymorph Selection of Zeolitic Imidazolate Frameworks via Kinetic and Thermodynamic Control. *ACS Cryst. Growth Des*. **2022**, 22, 4268-4276.
2. Hirai, K.; Ishikawa, H.; Chervy, T.; Hutchison, J.A; Uji-I, H. Selective crystallization via vibrational strong coupling. *Chemical Science* **2021**, *12*, 11986-11994. DOI: 10.1039/d1sc03706d.
3. Gayatri Kumari, Kolleboyina Jayaramulu, Tapas Kumar Maji, and Chandrabhas Narayana. Temperature Induced Structural Transformations and Gas Adsorption in the Zeolitic Imidazolate Framework ZIF-8: A Raman Study. *J.Phys.Chem.* A **2013**, 117, 11006-11012.
4. Markham, L. M.; Mayne, L. C.; Hudson, B. S. Resonance Raman Studies of Imidazole, Imidazolium, and Their Derivatives: The Effect of Deuterium Substitution. *J. Phys. Chem.* **1993**, 97, 10319− 10325.
5. Carter, D. A.; Pemberton, J. E. Raman Spectroscopy and Vibrational Assignments of 1- and 2 Methylimidazole. *J. Raman Spectrosc.* **1997**, 28, 939−946.
6. Mao, C.-J.; Hu, X.-W.; Song, J.-M.; Niu, H.-L.; Zhang, S.-Y. Synthesis of Zinc 1-(2-Pyridylazo)-2-Naphthol ($Zn(PAN)_2$) Nanobelts with Nonlinear Optical Property. *CrystEngComm* **2012**, 14, 6823−6826.
7. Guiling Luo, Ying Deng, Lin Zhu, Juan Liu, Bingxue Zhang, Yan Zhang, Wei Sun, and Guangjiu Li. Au-Co nanoparticles-embedded N-doped carbon nanotube hollow polyhedron modified electrode for electrochemical determination of quercetin. *Microchimica Acta* **2020**, 187, 546/1-9.
8. Shunsuke Tanaka, Kosuke Fujita, Yoshikazu Miyake, Manabu Miyamoto, Yasuhisa Hasegawa, Takashi Makino, Stijn Van der Perre, Julien Cousin Saint Remi, Tom Van

Assche, Gino V. Baron, and Joeri F.M. Denayer. Adsorption and Diffusion Phenomena in Crystal Size Engineered ZIF-8 MOF. *J. Phys. Chem. C* **2015**, 119, 28430-28439.
